# Characteristics and Clinical Implications of the Nasal Microbiota in Extranodal NK/T-Cell Lymphoma, Nasal Type

**DOI:** 10.3389/fcimb.2021.686595

**Published:** 2021-09-10

**Authors:** Zhuangzhuang Shi, Xin Li, Xinhua Wang, Lei Zhang, Ling Li, Xiaorui Fu, Zhenchang Sun, Zhaoming Li, Xudong Zhang, Mingzhi Zhang

**Affiliations:** ^1^Department of Oncology, The First Affiliated Hospital of Zhengzhou University, Zhengzhou, China; ^2^Lymphoma Diagnosis and Treatment Centre of Henan Province, Zhengzhou, China

**Keywords:** natural killer/T cell lymphoma, nasal microbiota, chronic rhinosinusitis, biomarker, 16S rRNA sequencing, nose-microbiota-UAT NKTCL axis

## Abstract

Natural killer/T cell lymphoma (NKTCL) most frequently affects the nasal cavity and upper aerodigestive tract (UAT) and is often mistaken for reactive disease processes, such as chronic rhinosinusitis (CRS). Recently, alterations of the nasal resident microbiota have been found in CRS. However, nasal microbial features in NKTCL have never been reported. This case-control study collected 46 NKTCL patients, 25 CRS patients and 24 matched healthy controls (HCs) to analyze nasal microbial profiles *via* 16S rRNA sequencing technology to improve our understanding of changes in the nasal microbiota in NKTCL. We found that alpha diversity was significantly decreased, while beta diversity was significantly increased in NKTCL compared with those in CRS and HCs. The genus *Corynebacterium* was significantly depleted in CRS and NKTCL *versus* that in HCs, while genus *Staphylococcus* was the most abundant in the NKTCL compared to that in the other two groups. The nasal microbial community was significantly different between UAT-NKTCL and non-UAT NKTCL patients. Importantly, based on a panel of taxa, excellent classification power with an AUC of 0.875 between UAT-NKTCL and CRS was achieved. Furthermore, the alpha diversity of the nasal microbiota was associated with several clinical covariates of NKTCL. Finally, PICRUSt analysis implicated an array of distinct functions in NKTCL that might be involved in the pathogenesis of the disease. In conclusion, the nasal microbial profile was unique in NKTCL. The nose-microbiota-UAT NKTCL axis represents a panel of promising biomarkers for clinical practice and contributes to revealing the potential pathogenesis of this malignancy.

## Introduction

Natural killer/T cell lymphoma (NKTCL) is a rare lymphoma subtype of peripheral T/NK-cell lymphoma that generally exhibits aggressive behavior and poor prognosis, with an elevated prevalence in Asia and South America (Aozasa et al., 2008; Haverkos et al., 2016). Although NKTCL constitutes a distinct category of systemic malignancy, it presents predominantly as a localized tumor of the upper aerodigestive tract (UAT), involving the nasal cavity and adjacent sites, with topical aggressive destruction (Liu et al., 2014). Additionally, tumors presenting outside the UAT whereas share identical histologic characteristics with UAT disease have also been classified as non-upper aerodigestive tract (NUAT) NKTCL, which has variable manifestations depending on the main site of involvement and typically exhibits a highly aggressive course (Au et al., 2009). Interestingly, low-grade NKTCL arising in the setting of chronic rhinosinusitis (CRS) has been reported in several studies (Tabanelli et al., 2014; Devins et al., 2018), and UAT-NKTCL is often mistaken as inflammatory reactive disease, such as CRS, based on the atypical initial clinical presentations, including nasal congestion, nasal discharge and epistaxis (Yen et al., 2012). However, early and accurate diagnosis with prompt treatment for NKTCL is vitally important considering its potentially devastating course and poor response to treatment, with additional sites of disease progression manifesting sometimes weeks or months after initial diagnosis (Yamaguchi et al., 2018).

The human nasal cavity is inhabited by a complex bacterial community which is mainly stable at the genus level, and bacterial colonization of the upper airways is a prerequisite for subsequent invasive disease (De Boeck et al., 2019). Advances in high-throughput genomic sequencing technology and the development of new methods for analyzing metagenomic data allow for a better understanding of the dynamic community of microbial flora that inhabit the human body in a relatively accessible and inexpensive manner. In recent years, accumulating studies have linked the nasal microbiota to a variety of diseases, including CRS, asthma, obstructive sleep apnea and granulomatosis with polyangiitis (Mahdavinia et al., 2018; Perez-Losada et al., 2018; Rhee et al., 2018; Wu et al., 2019). Furthermore, the human microbiota has tremendous potential to impact multiple human physiological functions including immune homeostasis (Belkaid and Hand, 2014). Dysbiosis, alterations of the microbial communities that lead to detrimental effects on the host, has been proposed to contribute to the genesis of several diseases of the lymphohematopoietic system, including Hodgkin lymphoma, gastric mucosa-associated lymphoid tissue (MALT) lymphoma, acute lymphoblastic leukemia and cutaneous T cell lymphoma (Cozen et al., 2013; Raderer et al., 2016; Rajagopala et al., 2016; Salava et al., 2020). Of them, *Helicobacter pylori* infection with gastric MALT lymphoma constitutes a paradigm for microbiota-driven malignancy (Raderer et al., 2016). Microbial community varies across different body sites, but much attention has been given to the gut microbiota, microbial communities in other body habitats, such as the nose, have also been involved in human health and disease (Kumpitsch et al., 2019). To the best of our knowledge, this is the first study to comprehensively estimate the nasal microbiota community in participants with NKTCL and compare it with that in both CRS patients and healthy controls (HCs) by 16S rRNA sequencing technology.

In this study, we mainly aimed to (1) identify the features of the nasal microbiome in NKTCL; (2) estimate the potential clinical value of the nasal microbiota in the management of the disease, including in distinguishing UAT-NKTCL from CRS in a timely manner and highlighting correlations between the nasal microbiota and several clinical covariates of NKTCL; and (3) finally, predict microbial functions associated with NKTCL using PICRUSt. Based on these analyses, we proposed that the nose-microbiota-UAT NKTCL axis, a bidirectional pathway of communication between the nasal microbiota and NKTCL, might be a fascinating avenue of research for this highly heterogeneous malignancy.

## Materials and Methods

### Study Subjects

Three groups (95 individuals) were enrolled in the present study. The demographic information and clinical features of all participants at the time of sampling are given in **Supplementary Table S1**. Among the patients recruited in this study, 46 patients with NKTCL (the NKT group) were identified by pathological diagnosis. The NKT group was further divided into UAT (presenting predominantly as a localized tumor of the UAT) and NUAT (presenting outside the UAT but have identical histologic profiles with UAT disease) subgroups based on the primary site of the tumor. The anatomical site of the primary lesion and the disease status of NKTCL patients were assessed by positron emission tomography-computed tomography, computed tomography (CT) or magnetic resonance image (MRI) scanning. The staging system was based on the Chinese Southwest Oncology Group and Asia Lymphoma Study Group ENKTL (CA) system (Hong et al., 2020). The risk stratification of patients with NKTCL was made by the prognostic index for natural killer lymphoma-Epstein-Barr virus (PINK-E) (Kim et al., 2016). Twenty-five patients with CRS (the CRS group) were identified by sinus CT/MRI scanning and nasal endoscopy. The healthy controls included 24 normal individuals selected among hospital researchers for the HC group with no prior cancer or acute/chronic rhinosinusitis diagnoses. Demographics were similar among the three groups. The participants included in our study were treated at our hospital between 2019 and 2020. The exclusion criteria were as follows: (1) patients with other malignancies, (2) individuals who received treatment with antibiotics or probiotics within two weeks, and (3) individuals with incomplete information. This research was carried out according to The Code of Ethics of the World Medical Association (Declaration of Helsinki), and was approved by the ethics review committee of the First Affiliated Hospital of Zhengzhou University. All the subjects provided informed consent for participation in this study.

### Sample Collection

Nasal samples were collected from the anterior nares using the method mentioned in the Manual of Procedures for the Human Microbiome Project (https://www.hmpdacc.org/hmp/doc/HMP_MOP_Version12_0_072910.pdf), with minor modifications. Briefly, with a twisting motion, approximately 2 centimeters of the mucosal surfaces of the anterior nares were gently rubbed with sterile flocked swabs, rotating 5 times against each wall of the anterior nares. The right and left sides are sampled successively and pooled together as a combined specimen. Immediately after swabbing, the collected samples were placed into 2 mL sterile tubes without enzyme, placed on ice after collection and stored at -80°C until the time of DNA extraction.

### DNA Extraction

Total DNA was extracted using the Swab Genomic DNA Kit (BGI-LH-302-03, China), according to the manufacturer’s instruction. After washing in 75% ethanol, DNA was resuspended in TE buffer and quantified with a Qubit Fluorometer by using Qubit dsDNA BR Assay kit (Invitrogen, USA), and the quality was checked by running aliquots on a 1% agarose gel.

### 16S Ribosomal RNA V4 Region Sequencing

To enable amplification of the V4 region of the bacterial 16S ribosomal RNA gene, the degenerate PCR primers, 515F (5’-GTGCCAGCMGCCGCGGTAA-3’) and 806R (5’GGACTACHVGGGTWTCTAAT-3’), as described by Caporaso et al. in 2011 (Caporaso et al., 2011), were designed. Both forward and reverse primers were tagged with Illumina adapter, pad and linker sequences. PCR enrichment was performed in a 50 μL reaction containing 30 ng of template, fusion PCR primers and PCR master mix. Thermal cycling consisted of an initial denaturation step at 95°C for 3 minutes, followed by 30 cycles of denaturation at 95°C for 45 seconds, annealing at 56°C for 45 seconds, and extension at 72°C for 45 seconds, with a final extension step at 72°C for 10 minutes. The PCR products were purified using Agencourt AMPure XP beads and eluted in elution buffer. The amplicon library for high-throughput sequencing was based on Illumina HiSeq 2500 platform, following the standard pipelines of Illumina, and generating 2 × 250 bp paired-end reads.

### Sequence Data Processing

Raw reads were filtered to remove adaptors and low-quality and ambiguous bases, and then paired-end reads were added to tags by the Fast Length Adjustment of Short reads program (FLASH, v1.2.11) to obtain the tags. The processed tags were clustered into operational taxonomic units (OTUs) at the commonly used 97% similarity threshold using UPARSE software (v7.0.1090) and chimera sequences were compared with the Gold database using UCHIME (v4.2.40). Then, the OTUs were assigned to taxa by matching to the Greengenes database v201305 with QIIME v1.8.0. Species with relative abundances lower than 0.5% in all samples and those not noted at the corresponding classification level were merged into “Others”. Venn plots of OTUs were plotted with the R package “VennDiagram” v3.1.1. Alpha and beta diversity were estimated by MOTHUR (v1.31.2) and QIIME (v1.8.0) at the OTU level, respectively. LEfSe clustering and linear discriminant analysis (LDA) analysis were conducted by LEfSe. Significant species or functions were determined by R (v3.4.1) based on the Wilcoxon test. KEGG functions were predicted using PICRUSt software.

### Statistical Analysis

All statistical analyses were carried out using R (v3.6.1) for Windows. We compared continuous variables by the Wilcoxon rank sum test (unless otherwise specified) between both groups and Fisher’s exact test for categorical variables. Receiver operating characteristic (ROC) curves were obtained, and the area under the curve (AUC) was used to designate the ROC effect. P < 0.05 was considered statistically significant. All *P* values were two-sided. *P < 0.05, **P < 0.01, ***P < 0.001.

## Results

### Biodiversity of the Nasal Microbiota in NKTCL and CRS Patients and Healthy Controls

**Figure 1:**
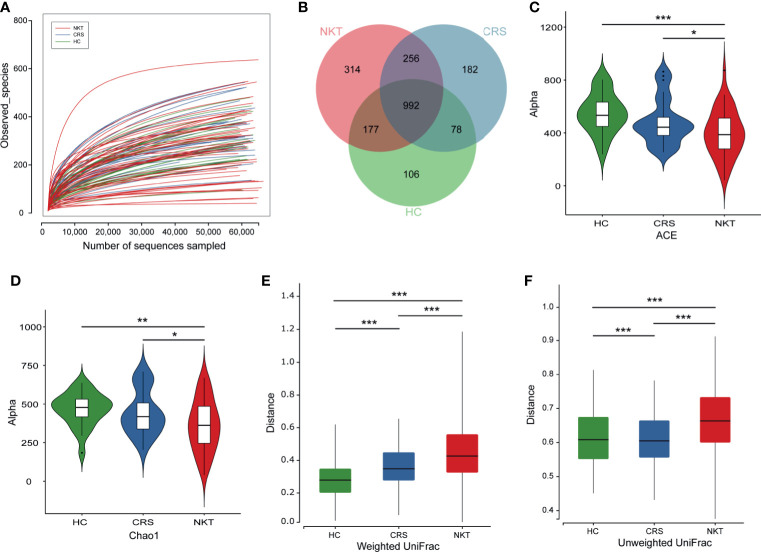
Biodiversity of the nasal microbiota in the indicated groups. **(A)** Rarefaction curves of samples in the NKT (n = 46), CRS (n = 25) and HC groups (n = 24). **(B)** A Venn diagram displaying the overlap of OTUs among groups. Comparison of the microbial alpha diversity among groups using *Student’s t* test. **(C)** For the ACE index, *P* = 0.0481, NKT *vs* CRS; *P* < 0.001, NKT *vs* HC; **(D)** for the Chao1 index, *P* = 0.0353, NKT *vs* CRS; *P* = 0.0023, NKT *vs* HC. For beta diversity as measured by **(E)** the weighted UniFrac, *P* = 4.42e-18, NKT *vs* CRS; *P* =8.24e-47, NKT *vs* HC; *P* = 7.67e-11 CRS *vs* HC. **(F)** For the unweighted UniFrac index, *P* = 1.81e-28, NKT *vs* CRS; *P* = 1.49e-23, NKT *vs* HC; *P* = 1.05e-6 CRS *vs* HC. NKT, natural killer/T cell lymphoma; CRS, chronic rhinosinusitis; HC, healthy control; OTUs, operational taxonomy units; *P < 0.05, **P < 0.01, ***P < 0.001.

Rarefaction analysis showed that the observed species basically approached saturation in each sample, which suggested that the sequencing data were sufficient, with very few new species undetected (**Figure 1A**). In addition, a Venn diagram exhibiting the overlaps between groups indicated that 314 of the total 2105 OTUs were unique to NKTCL, and 1248 of 1999 OTUs were shared between the CRS and NKT groups, while 992 of 2105 OTUs were shared among the three groups (**Figure 1B**). Alpha diversity metrics, as estimated by the ACE (**Figure 1C**) and Chao1 (**Figure 1D**) indices, showed that the NKT group had the lowest biodiversity compared with the CRS (*P* = 0.0481 and 0.0353, *t* test) and healthy controls (*P* < 0.001 and *P* = 0.0023, *t* test). There were no significant differences in alpha diversity between patients with CRS and healthy individuals, and no statistical differences were found when comparing Shannon and Simpson indices between groups (**Supplementary Figure S1**). Regarding nasal bacterial community structure, beta diversity calculated by the weighted UniFrac (**Figure 1E**) and unweighted UniFrac (**Figure 1F**) indices exhibited that the homogeneity of the nasal microbial communities in NKTCL patients was lower than that of the communities in the other two groups of subjects, indicating a unique nasal microbiota in NKTCL.

### Phylogenetic Features of the Nasal Microbiota in NKTCL

**Figure 2:**
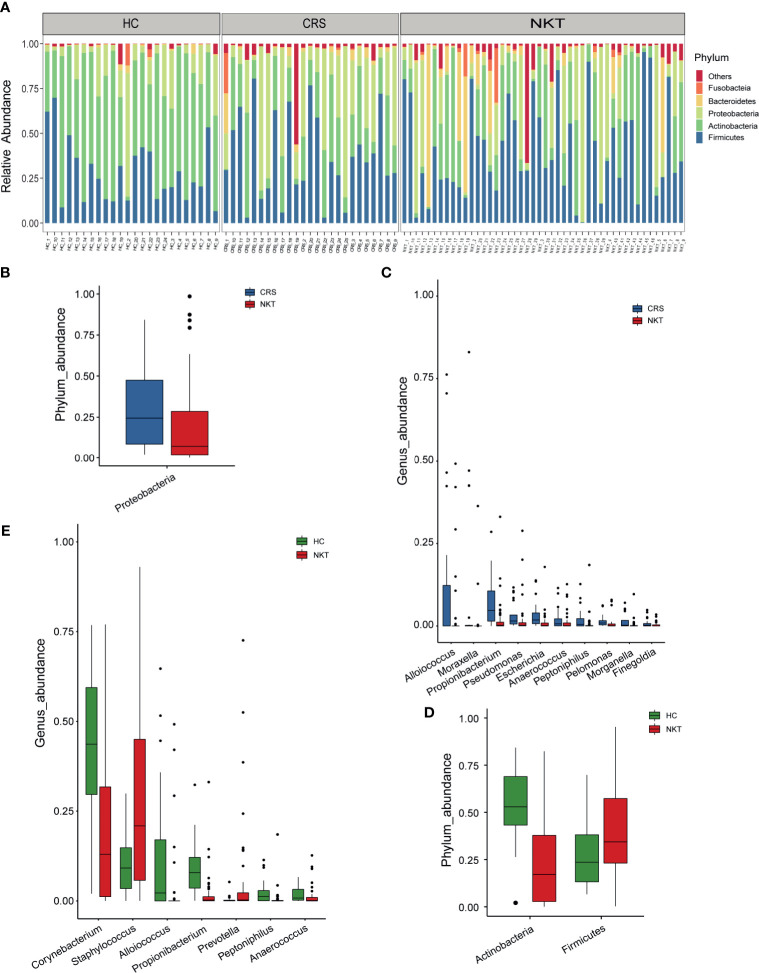
Phylogenetic features of nasal microbiota among patients with NKTCL (n = 46), patients with CRS (n = 25) and HCs (n = 24). Only phyla or genera with relative abundances > 0.5% are shown. **(A)** The bacterial phylum composition of each specimen (n = 95). The significantly depleted microbial community members at the **(B)** phylum level and **(C)** genus level in the NKT group *versus* the CRS group. The significantly differentially abundant microbial community members at the **(D)** phylum level and **(E)** genus level in the NKT group *versus* the HC group. The box presents the 95% CI; the line inside denotes the median. NKT, natural killer/T cell lymphoma; CRS, chronic rhinosinusitis; HC, healthy control.

The taxonomic composition and dysregulations of the nasal microbiota among groups were analyzed *via* 16S sequencing. At the phylum level, the dominant taxa consisted of *Firmicutes*, *Actinobacteria*, *Proteobacteria*, *Bacteroidetes* and *Fusobacteria* in all groups (**Figure 2A** and **Supplementary Figure S2A**). The average composition of the microbial community at the phylum, family and genus levels is displayed in the **Supplementary Figure S2**. Compared with that in the CRS group, the phylum *Proteobacteria* was significantly depleted in the NKT group (**Figure 2B**, *P* = 0.009815). Correspondingly, 10 genera including *Alloiococcus*, *Moraxella* and *Propionibacterium*, were enriched in the CRS group *versus* the NKT group (**Figure 2C**, all *P* < 0.05, **Supplementary Table S2**). Compared with those in healthy controls, the phylum *Actinobacteria* was significantly depleted, and *Firmicutes* was significantly enriched in NKTCL patients (**Figure 2D**, *P* < 0.001 and *P* = 0.048438, respectively). Six genera, at the genus level, including *Corynebacterium*, *Alloiococcus* and *Propionibacterium*, were significantly depleted, while *Staphylococcus* was enriched in the NKT group *versus* the control group (**Figure 2E**, all *P* < 0.05, **Supplementary Table S3**). Intriguingly, from the HC group to the CRS group to the NKT group, *Staphylococcus* was gradually enriched although no significant difference was found between CRS with the other two groups (**Supplementary Figure S3C**). In addition, bacterial differences at the phylum and genus levels between CRS patients and HCs were compared and are exhibited in **Supplementary Figure S4** and **Supplementary Tables S4.1, 4.2**, respectively. Of them, phylum *Proteobacteria* was significantly enriched (**Supplementary Figure S4A**, *P* = 0.007585) whereas genus *Corynebacterium* was significantly depleted (**Supplementary Figure S4B**, *P* < 0.001) in the CRS group compared with that in HC group, and microbial dysregulations in family level between groups are shown in **Supplementary Figure S5** and **Supplementary Tables S5.1-5.3**.

### Distinct Nasal Microbiota Profiles in NUAT-NKTCL

**Figure 3:**
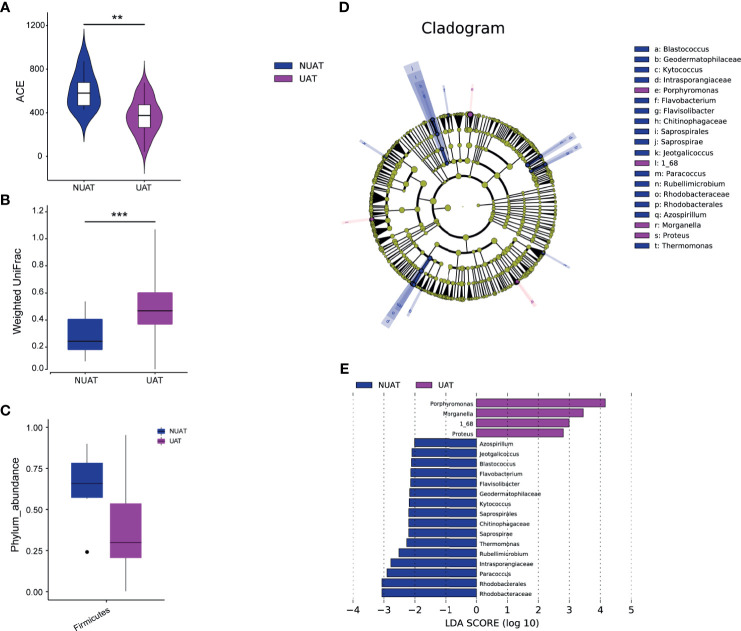
Unique nasal microbiota profiles in patients with NUAT-NKTCL. Comparison of the nasal microbial **(A)** alpha diversity (ACE index), *P* = 0.00323, Wilcoxon test, and **(B)** beta diversity, *P* < 0.001, weighted UniFrac index, between the NUAT and UAT groups. **(C)** The abundance of the phylum *Firmicutes* was significantly increased in the NUAT (n = 6) *versus* UAT (n = 40) patients (*P* = 0.0415). LEfSe for the nasal microbiota in the NUAT and UAT groups. **(D)** The cladogram shows differences in microbial taxa between NUAT and UAT patients. Letters refer to differentially abundant taxa. **(E)** The histogram represents linear discriminant analysis (LDA) scores of bacteria with significantly different abundances (LDA > 2) between the two groups, as represented by different colors. The taxa with LDA > 2 are shown. NUAT, non-upper aerodigestive tract; UAT, upper aerodigestive tract; LDA, linear discriminant analysis; LEfSe, LDA effect size; **P < 0.01, ***P < 0.001.

The nasal microbial community in NUAT-NKTCL was significantly different from that in UAT-NKTCL. The bacterial community profiles for patients with NUAT-NKTCL had a higher alpha diversity (**Figure 3A**, *P* = 0.00323, measured by ACE index) and were more homogeneous (**Figure 3B**, *P* < 0.001, measured by the weighted UniFrac index) than those for patients with UAT-NKTCL, but there was no significant difference in the Chao1, Shannon, Simpson and unweighted UniFrac indices (**Supplementary Figure S6**). At the phylum level, patients with NUAT-NKTCL showed a higher prevalence rate of *Firmicutes* than patients with UAT-NKTCL (**Figure 3C**, *P* = 0.041497). Additionally, the greatest difference in microbial structure between NUAT-NKTCL and UAT-NKTCL patients was displayed using the LEfSe method (**Figure 3D**). More specifically, 16 taxa, including *Rhodobacteraceae*, *Rhodobacterales*, *Paracoccus*, *Intrasporangiaceae*, *Rubellimicrobium*, *Thermomonas*, *Saprospirae*, *Chitinophagaceae*, *Saprospirales*, *Kytococcus*, *Geodermatophilaceae*, *Flavisolibacter*, *Flavobacterium*, *Blastococcus*, *Jeotgalicoccus* and *Azospirillum* were enriched, whereas 4 taxa, including *Porphyromonas*, *Morganella*, *1_68* and *Proteus* were depleted in NUAT-NKTCL *versus* UAT-NKTCL patients based on LDA selection (**Figure 3E**).

### Novel Microbial Biomarkers for UAT-NKTCL Diagnosis

**Figure 4:**
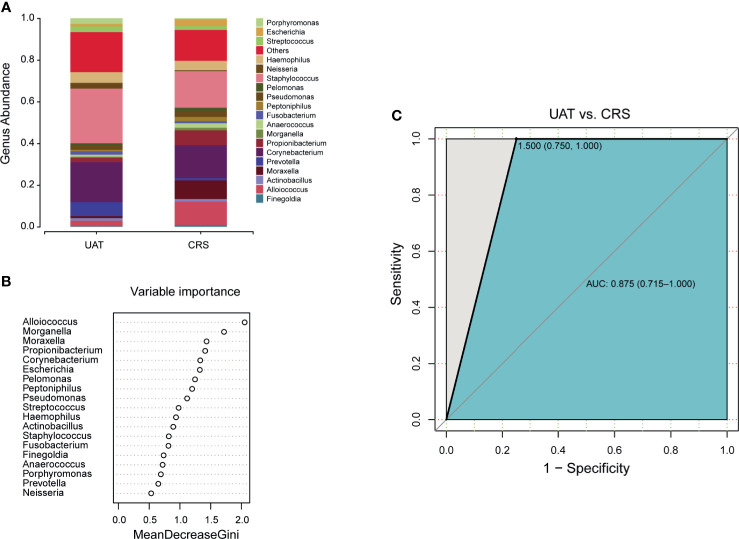
Panels of novel microbial biomarkers for UAT-NKTCL diagnosis. **(A)** Composition of the nasal microbiota at the genus level between the UAT and CRS groups (only genera with relative abundances > 0.5% are shown), and **(B)** these taxa are ranked from the top to bottom by decreasing Gini index scores determined from the random forest algorithm trained to distinguish the 2 cohorts. Importantly, these genus markers in combination might be used to distinguish UAT-NKTCL from CRS, **(C)** achieving an AUC of 0.875 (95% CI: 0.715 to 1.0). UAT, upper aerodigestive tract; CRS, chronic rhinosinusitis; AUC, area under the curve; 95% CI, 95% confidence interval.

The initial clinical manifestation of UAT-NKTCL bears a remarkable resemblance to the manifestation of CRS, and most UAT-NKTCL patients have a preexisting history of CRS. Based on the genera with relative abundances greater than 0.5% between the UAT and CRS groups (**Figure 4A**), a random forest machine learning algorithm was applied to search for a highly fitting model discriminating the two cohorts. These taxa are ranked from top to bottom by decreasing Gini index scores (**Figure 4B**). As Gini index scores quantify the strength of each respective predictor, the best predictors of UAT group are at the top of the plot. These nasal microbial marker panels in combination achieved an AUC value of 0.875 with a 95% CI of 0.715 to 1.0 between UAT and CRS (**Figure 4C**). The differential diagnostic models between the UAT and HC groups and between the CRS and HC groups are shown in **Supplementary Figures S7****,** **S8**, respectively.

### Associations of the Nasal Microbiota With Disease Status and NKTCL Prognosis Indicators

**Figure 5:**
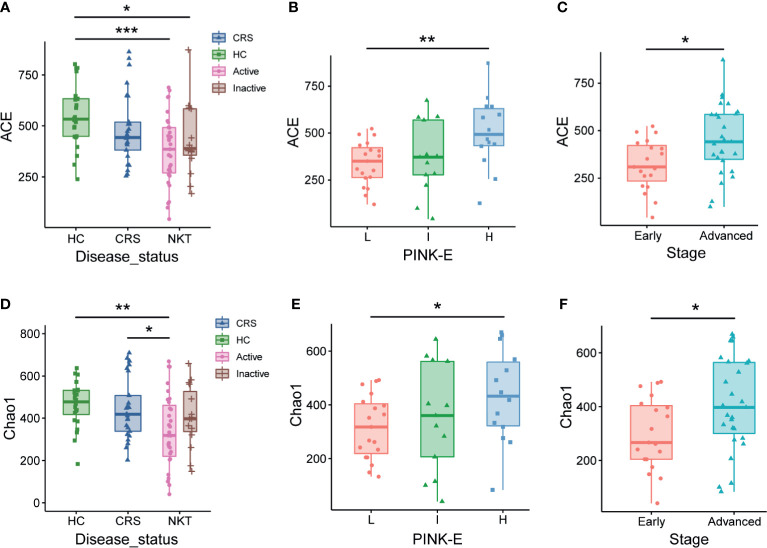
Associations of the nasal microbiota with disease status and prognosis indicators of NKTCL. Alpha diversity of the nasal microbiota, as measured by the ACE **(A–C)** and Chao1 **(D–F)** index, in active and inactive status; low risk, intermediate risk, and high risk based on PINK-E; and early and advanced stage NKTCL patients. For the ACE index, **(A)** *P* = 0.00051, Active *vs* HC; *P* = 0.03028, Inactive *vs* HC; **(B)** *P* = 0.00545, L *vs* H; **(C)** *P* = 0.01697, Early *vs* Advanced. For the Chao1 index, **(D)** *P* = 0.00308, Active *vs* HC; *P* = 0.02525, Active *vs* CRS; **(E)** *P* = 0.03172, L *vs* H; **(F)** *P* = 0.0331, Early *vs* Advanced. HC, healthy control; CRS, chronic rhinosinusitis; NKT, natural killer/T cell lymphoma; L, low risk; I, intermediate risk; H, high risk; PINK-E, prognostic index for natural killer lymphoma-Epstein-Barr virus; *P < 0.05, **P < 0.01, ***P < 0.001.

When evaluating the associations between alpha diversity and clinical covariates of NKTCL, we found that the nasal microbial communities in patients with NKTCL in remission were more similar to those in healthy controls and CRS patients than to those in participants with active NKTCL. Specifically, alpha diversity analysis showed significantly reduced ACE (**Figure 5A**, *P* = 0.00051, Active *vs* HC) and Chao1 (**Figure 5D**, *P* = 0.02525 Active *vs* CRS; *P* = 0.00308, Active *vs* HC) indices of nasal bacterial populations of patients with active disease compared with that of the nasal bacterial populations of CRS patients or HCs. Interestingly, compared with patients at intermediate and high risk based on PINK-E, NKTCL patients at low risk had the lowest ACE (**Figure 5B**, *P* = 0.00545, low risk *vs* high risk) and Chao1 (**Figure 5E**, *P* = 0.03172, low risk *vs* high risk) indices. In addition, early-stage patients with NKTCL had lower ACE and Chao1 indices than advanced patients (**Figures 5C, F**, *P* = 0.01697 and 0.0331, respectively). Comparisons by the Shannon and Simpson indices are shown in **Supplementary Figure S9**.

### Functional Prediction of Microbial Genes Associated With NKTCL

**Figure 6:**
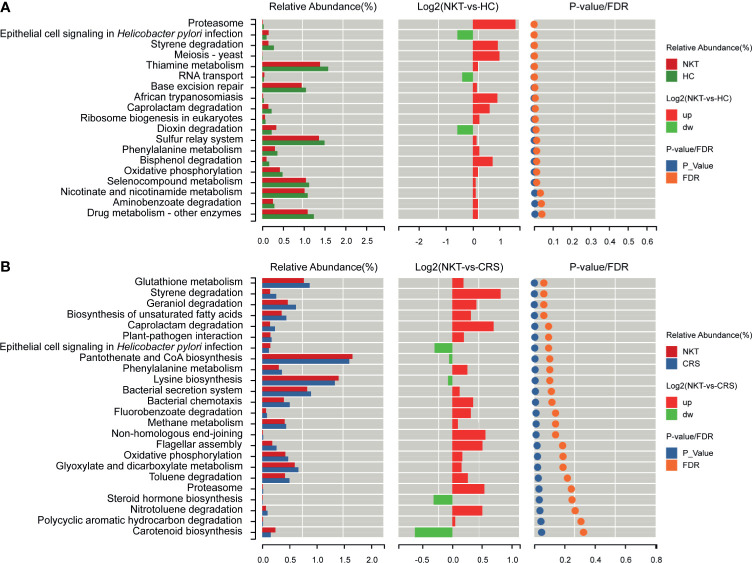
Functional prediction of microbial genes associated with NKTCL by PICRUSt. **(A)** Compared with those in HCs, based on LDA selection, 3 predicted microbial functions, including epithelial cell signaling in *Helicobacter pylori* infection, RNA transport and dioxin degradation, were enriched, while 16 functions, including proteasome, styrene degradation and meiosis-yeast, were reduced in NKTCL patients. **(B)** Compared with those in CRS, 5 predicted microbial functions, including epithelial cell signaling in *Helicobacter pylori* infection, pantothenate and CoA biosynthesis and lysine biosynthesis increased, whereas 19 functions, including glutathione metabolism, styrene degradation and geraniol degradation decreased in NKTCL, but no significantly different function was found after FDR adjustment. NKT, natural killer/T cell lymphoma; CRS, chronic rhinosinusitis; HC, healthy control; PICRUSt, Phylogenetic Investigation of Communities by Reconstruction of Unobserved States; LDA, linear discriminant analysis; FDR, false discovery rate.

Compared with those in HCs, based on LDA selection, 3 predicted microbial functions, including epithelial cell signaling in *Helicobacter pylori* infection, RNA transport and dioxin degradation, were enriched, while 16 functions, including proteasome, styrene degradation and meiosis-yeast, were depleted in NKTCL patients (**Figure 6A** and **Supplementary Table S6**). Moreover, compared to those in CRS, 5 functions, including epithelial cell signaling in *Helicobacter pylori* infection, pantothenate and CoA biosynthesis and lysine biosynthesis, increased, whereas 19 functions, including glutathione metabolism, styrene degradation and geraniol degradation, decreased in NKTCL patients, but there was no significant difference after false discovery rate (FDR) adjustment (**Figure 6B** and **Supplementary Table S7**), suggesting that these microbial gene functions are highly relevant to the pathogenesis of NKTCL.

## Discussion

NKTCL, featured by angioinvasion, angiodestruction, necrosis and strong linkage to *Epstein-Barr virus* (EBV), is a rare but highly aggressive disease commonly involving the nasal cavity and UAT (Li et al., 2013; Tse et al., 2019). Recently, nasal microbiome has been reported to be a useful tool and biomarker in the clinical management of multiple diseases (Mahdavinia et al., 2018; Perez-Losada et al., 2018; Rhee et al., 2018; Hsiao et al., 2021). Nevertheless, the role of the nasal microbes in the complex interplay between the host and environment is far from understood. In the pilot study, we assessed nasal microbial flora in NKTCL patients, CRS patients and control subjects to identify nasal microbiota patterns by next-generation sequencing, for the sake of providing new insights into the role of the nasal microbiota in the pathogenesis and clinical translations of NKTCL.

This study, for the first time, illustrated nasal microbial profiles in an NKTCL cohort by HiSeq sequencing. The alpha diversity, as measured by the ACE and Chao1 indices, of the nasal microbiota in NKTCL was significantly decreased, while the beta diversity was significantly increased, but there was no significant difference in alpha diversity between CRS patients and control subjects, which is consistent with previously published data (Ramakrishnan et al., 2015; Mahdavinia et al., 2018). Furthermore, five phyla, among the three groups, constituted the predominant nasal microbiota, including *Firmicutes*, *Actinobacteria*, *Proteobacteria*, *Bacteroidetes* and *Fusobacteria*, in this study. Of note, previous studies revealed that the phylum *Proteobacteria* was significantly enriched in patients with CRS and asthma (Choi et al., 2014; Fazlollahi et al., 2018; Ta et al., 2018). Furthermore, a bloom of *Proteobacteria* in the gut reflects a compromised ability to maintain a balanced microbial community, which can further facilitate inflammation or invasion by exogenous pathogens (Shin et al., 2015). In this work, we found that microbial disturbances presented a significant increase in the relative abundance of the phylum *Proteobacteria* in the CRS group compared with that in both the NKT and HC groups, but there was no significant difference between the NKT and HC groups. Accordingly, the long-term inflammatory stimulation by *Proteobacteria* in CRS is likely to engage in the malignant transformation of lymphocytes, which might be involved in the genesis of NKTCL but not in the maintenance of the disease once malignant transformation is initiated.

Researchers previously suggested that the genus *Corynebacterium* is crucial for maintaining a stable and sustained microbial pattern in the nasal cavity in healthy infants by releasing antibacterial free fatty acids from human triacylglycerols (Bomar et al., 2016; Ta et al., 2018). Most importantly, Ramakrishnan, Vijay R. et al. found that an increased abundance of *Corynebacterium* at the time of endoscopic sinus operation was predictive of better surgical outcomes (Ramakrishnan et al., 2015). As shown by our data, compared with that in healthy individuals, the genus *Corynebacterium* was significantly depleted in both the CRS and NKT subjects. Thus, decreased colonization of *Corynebacterium* might remove a protective element, potentially contributing to the pathogenesis and progression of NKTCL.

A variety of studies have reported a higher rate of *Staphylococcus aureus* nasal colonization in patients with granulomatosis with polyangiitis than in the general population, and chronic nasal carriage has been related to an increased risk of disease relapse (Wagner et al., 2019). Moreover, it has been demonstrated that cutaneous T cell lymphoma patients are frequently colonized with *S. aureus* expressing staphylococcal enterotoxins (SEs) that induce crosstalk between malignant and benign T cells, leading to Stat3-mediated interleukin-10 production by malignant T cells, which has an important role in driving immune dysregulation and severe immunodeficiency (Krejsgaard et al., 2014). Importantly, in this study, from the HC group to the CRS group to the NKT group, the genus *Staphylococcus* was gradually enriched and had a significantly higher abundance in the NKT group than in the HC group. It could thus be speculated that *Staphylococcus* might be responsible for the suppression of NKTCL patients’ cellular immunity and antitumor responses. Of interest, Ramsey, M. M. et al. reported that *S. aureus* shifts from virulence toward a commensal status when exposed to commensal *Corynebacterium* species (Ramsey et al., 2016), suggesting that enriched *Staphylococcus* and depleted *Corynebacterium* may be an important etiological factor for NKTCL. Furthermore, the genera *Propionibacterium*, *Alloiococcus*, *Peptoniphilus* and *Anaerococcus* were significantly depleted in NKTCL patients compared with those in both CRS patients and HCs. Altogether, these findings indicated that nasal microbial dysbiosis might be intimately engaged in the pathogenesis of NKTCL, and further investigations into the complex mechanisms of nasal cavity bacterial-mediated lymphomagenesis are warranted.

NKTCL can be well divided into two clinical subtypes: UAT-NKTCL and NUAT-NKTCL (Liu et al., 2014). Although both subtypes share identical histologic features, it has long been debated whether they are the same disease in consideration of obviously different clinical characteristics and outcomes between the two cohorts (Yeh et al., 1999; Li et al., 2013). In the NKTCL patients of our study, we observed significantly different ACE and weighted UniFrac indices, as well as configurations between patients with UAT-NKTCL and those with NUAT-NKTCL. Compared with that in patients with UAT-NKTCL, the phylum *Firmicutes* was significantly enriched in patients with NUAT-NKTCL. Furthermore, great differences in microbial composition between UAT-NKTCL and NUAT-NKTCL were displayed *via* LEfSe analysis. Even so, it is prudent to draw the conclusion that the two clinical subtypes of NKTCL belong to different diseases due to the small sample size (n=6) of NUAT-NKTCL.

Furthermore, accurate and prompt diagnosis of NKTCL, especially UAT-NKTCL, can be difficult because of nonspecific clinical manifestations, small biopsy samples, tissue necrosis, and background cellular reaction (Li et al., 2013; Miyake et al., 2014). Accordingly, our study found that based on a panel of nasal microbes with abundances greater than 0.5%, excellent classification power with an AUC of 0.875 between UAT-NKTCL and CRS was achieved. Considering the easily operable and noninvasive specimen and cost-effectiveness merits, the specific alterations of the nasal microbiota might provide a new biomarker for UAT-NKTCL diagnosis with high accuracy, which requires validation in a larger cohort.

When examining the relationships between the alpha diversity of the nasal microbiome and several clinical covariates of patients with NKTCL, we found that alpha diversity was closely associated with disease status, with the lowest ACE and Chao1 indices for active NKTCL patients, suggesting that alpha diversity might be a promising biomarker for monitoring the disease. Interestingly, the ACE and Chao1 indices gradually increased in NKTCL patients from low risk to intermediate risk and to high risk, and patients in an advanced stage had a significantly increased ACE and Chao1 indices compared with that of early-stage patients with NKTCL. In consideration of more widespread systemic manifestations, not just those confined to the nasal cavity, of NKTCL patients in high-risk and advanced stages, the high-risk and advanced patients might suffer relatively little nasal microbial disorders compared with low-risk and localized disease patients. Although no significant difference has been found between groups in Shannon and Simpson indices, these results hinted that the changed nasal microbiota may represent a useful tool in risk stratification and staging of the disease, and the potential influences of diverse therapeutic regimens on nasal microbiota merit further evaluation.

Distinct microbial communities generate different microbial products and serve unique functions, thereby contributing to the pathogenesis and development of a variety of diseases once the subtle microbiome balance fails. In our study, by PICRUSt analysis based on the FDR, a range of distinct microbial functions have been revealed in NKTCL patients in comparison with those in healthy individuals. Moreover, although we did not observe significant functional differences between patients with NKTCL and patients with CRS after FDR adjustment, these differences might emerge as the sample size increases. Importantly, these results of microbial functions might provide beneficial insights into the etiology and development of NKTCL, which implied that the two-way nose-microbiota-UAT NKTCL axis might be a potential target to prevent the development of NKTCL.

Among the limitations pertinent to this study we should point out that the observational nature of our study cannot illustrate whether alterations in nasal microbes are a cause or effect of NKTCL. Therefore, more comprehensive data regarding host immune status and further germ-free mouse or microbial transplantation to diseased animal models along with omics techniques (e.g., metagenomics, metabolomics, metatranscriptomics and metaproteomics) are essential to validate the potential causative relationship and the underlying mechanisms between the nasal microbiota and the pathogenesis of NKTCL. Additionally, well-designed prospective studies recruiting larger treatment-naive populations with longitudinal follow-up are needed to confirm the clinical value of the nose-microbiota-UAT NKTCL axis as a novel biomarker or target in the diagnosis and management of the disease.

## Conclusions

In summary, the present study has shed light on the profiles of microbial communities living in the anterior nares of patients with NKTCL and identified several microbial biomarkers for the clinical management of the disease. Research into the interactions between specific nasal microbiome features and the pathogenesis of NKTCL could ultimately advance our understanding of this malignancy and improve current diagnostic markers and treatment options of the disease for custom-fit precision medicine.

## Data Availability Statement

The 16S rRNA sequencing data reported in this paper are available from NCBI SRA database under BioProject accession number PRJNA733573. All other data are available in this manuscript including its supplementary files, or from the corresponding author upon reasonable request. https://www.ncbi.nlm.nih.gov/bioproject/PRJNA733573.

## Ethics Statement

This study was approved by the Ethics Review Committee of the First Affiliated Hospital of Zhengzhou University, and the study was performed in accordance with the Helsinki Declaration and rules of good clinical practice. All the participants provided written informed consents after the study protocol was fully explained.

## Author Contributions

MZ and ZZS designed the study and analyzed data. ZZS, XL, and XW wrote the manuscript. ZZS, XL, XW, LZ, and LL collected data. ZZS, LZ, LL, and XF performed research. MZ, ZZS, ZL, XF, and XZ interpreted data. XL, XW, ZCS, and ZL contributed technical and material support. All authors contributed to the article and approved the submitted version.

## Funding

This work was supported by the National Natural Science Foundation of China (Grant No. 81970184) and the National Science and Technology Major Project of China (Grant No. 2020ZX09201-009).

## Conflict of Interest

The authors declare that the research was conducted in the absence of any commercial or financial relationships that could be construed as a potential conflict of interest.

## Publisher’s Note

All claims expressed in this article are solely those of the authors and do not necessarily represent those of their affiliated organizations, or those of the publisher, the editors and the reviewers. Any product that may be evaluated in this article, or claim that may be made by its manufacturer, is not guaranteed or endorsed by the publisher.

## Abbreviations

NKTCL/NKT, natural killer/T cell lymphoma; UAT, upper aerodigestive tract; NUAT, non-upper aerodigestive tract; CRS, chronic rhinosinusitis; HCs, healthy controls; MALT, mucosa-associated lymphoid tissue; CT, computed tomography; MRI, magnetic resonance image; PINK-E, prognostic index for natural killer lymphoma-Epstein-Barr virus; OTUs, operational taxonomic units; ROC, receiver operating characteristic; AUC, area under the curve; LDA, linear discriminant analysis; LEfSe, LDA effect size; CI, confidence interval; L, low risk; I, intermediate risk; H, high risk; PICRUSt, Phylogenetic Investigation of Communities by Reconstruction of Unobserved States; FDR, false discovery rate; EBV, *Epstein-Barr virus*.
