# Supplementary material for: Characteristics and Clinical Implications of the Nasal Microbiota in Extranodal NK/T-Cell Lymphoma, Nasal Type
Source: Front Cell Infect Microbiol. 2021 Sep 10;11:686595. doi: 10.3389/fcimb.2021.686595 (PMC8461088; doi:10.3389/fcimb.2021.686595)
Supplement: Supplementary file 2 [file Image_1.pdf]

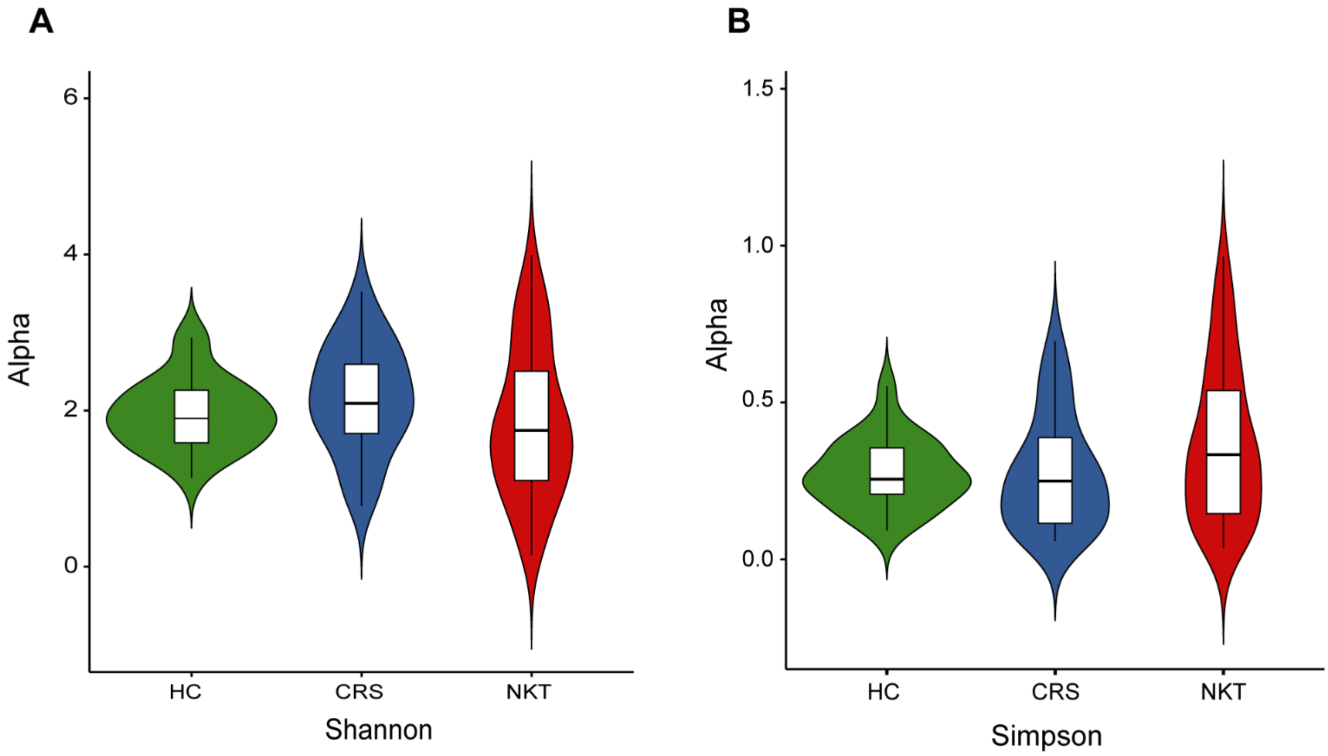

Figure S1 Alpha diversity metrics estimated by the (A) Shannon and (B) Simpson indices. No significant difference was found among groups (all $P > 0.05$). Abbreviations: NKT, natural killer/T cell lymphoma; CRS, chronic rhinosinusitis; HC, healthy control.
