# Supplementary material for: Characteristics and Clinical Implications of the Nasal Microbiota in Extranodal NK/T-Cell Lymphoma, Nasal Type
Source: Front Cell Infect Microbiol. 2021 Sep 10;11:686595. doi: 10.3389/fcimb.2021.686595 (PMC8461088; doi:10.3389/fcimb.2021.686595)
Supplement: Supplementary file 3 [file Image_2.pdf]

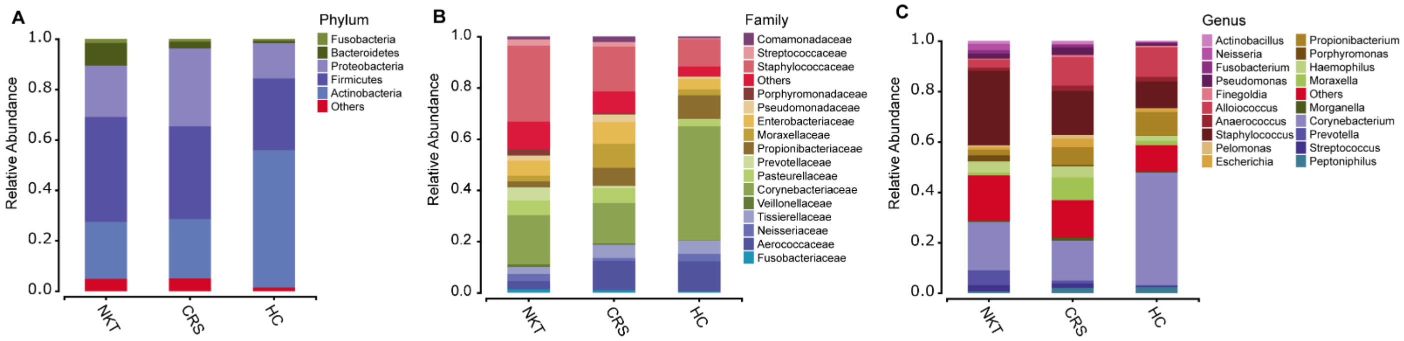

Figure S2 Average composition of bacterial community at the (A) phylum, (B) family and (C) genus levels. Bar charts depict the percentage of relative abundance of prominent phyla, families or genera with abundances greater than 0.5%. Abbreviations: NKT, natural killer/T cell lymphoma; CRS, chronic rhinosinusitis; HC, healthy control.
