# Supplementary material for: Characteristics and Clinical Implications of the Nasal Microbiota in Extranodal NK/T-Cell Lymphoma, Nasal Type
Source: Front Cell Infect Microbiol. 2021 Sep 10;11:686595. doi: 10.3389/fcimb.2021.686595 (PMC8461088; doi:10.3389/fcimb.2021.686595)
Supplement: Supplementary file 4 [file Image_3.pdf]

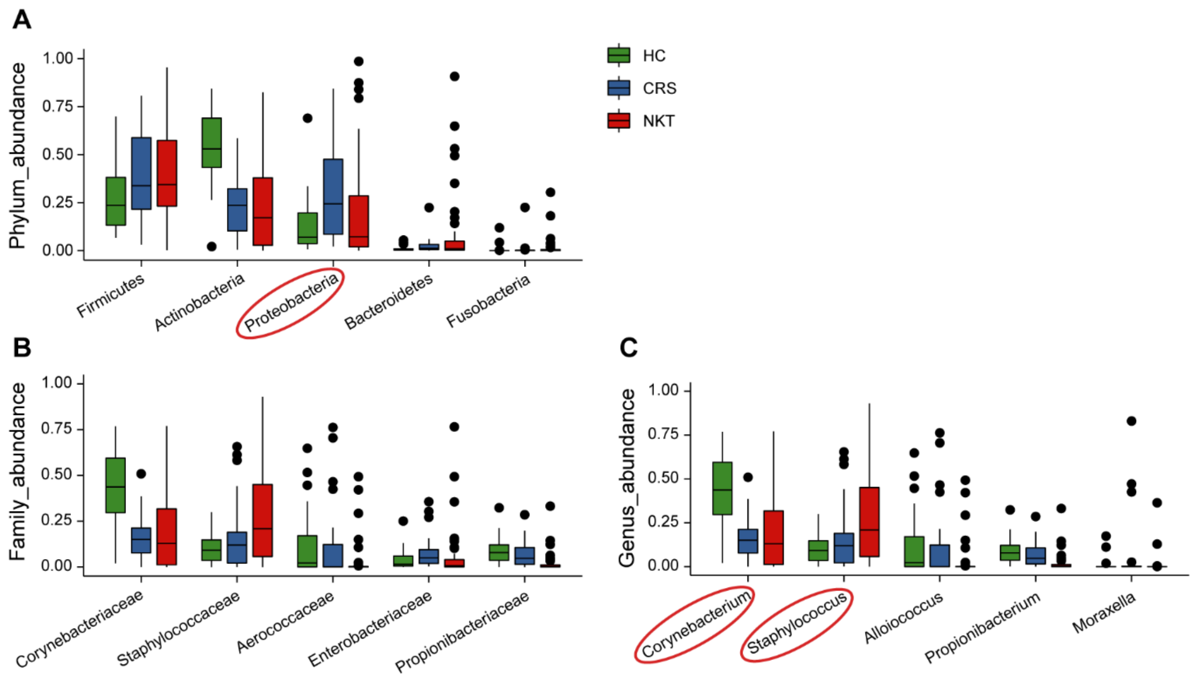

Figure S3 Comparison of the top 5 taxa of the nasal microbial composition among the three groups at the (A) phylum level, for the phylum *Proteobacteria*, $P = 0.009815$, CRS vs NKT; $P = 0.007585$, CRS vs HC; $P = 0.886851$, NKT vs HC; (B) family level, and (C) genus level, for the genus *Corynebacterium*, $P < 0.001$, HC vs NKT and HC vs CRS; $P = 0.740598$, NKT vs CRS; for the genus *Staphylococcus*, $P = 0.016104$, NKT vs HC; $P = 0.105406$, NKT vs CRS; $P = 0.638355$, HC vs CRS. Abbreviations: NKT, natural killer/T cell lymphoma; CRS, chronic rhinosinusitis; HC, healthy control.
