# Supplementary material for: Characteristics and Clinical Implications of the Nasal Microbiota in Extranodal NK/T-Cell Lymphoma, Nasal Type
Source: Front Cell Infect Microbiol. 2021 Sep 10;11:686595. doi: 10.3389/fcimb.2021.686595 (PMC8461088; doi:10.3389/fcimb.2021.686595)
Supplement: Supplementary file 5 [file Image_4.pdf]

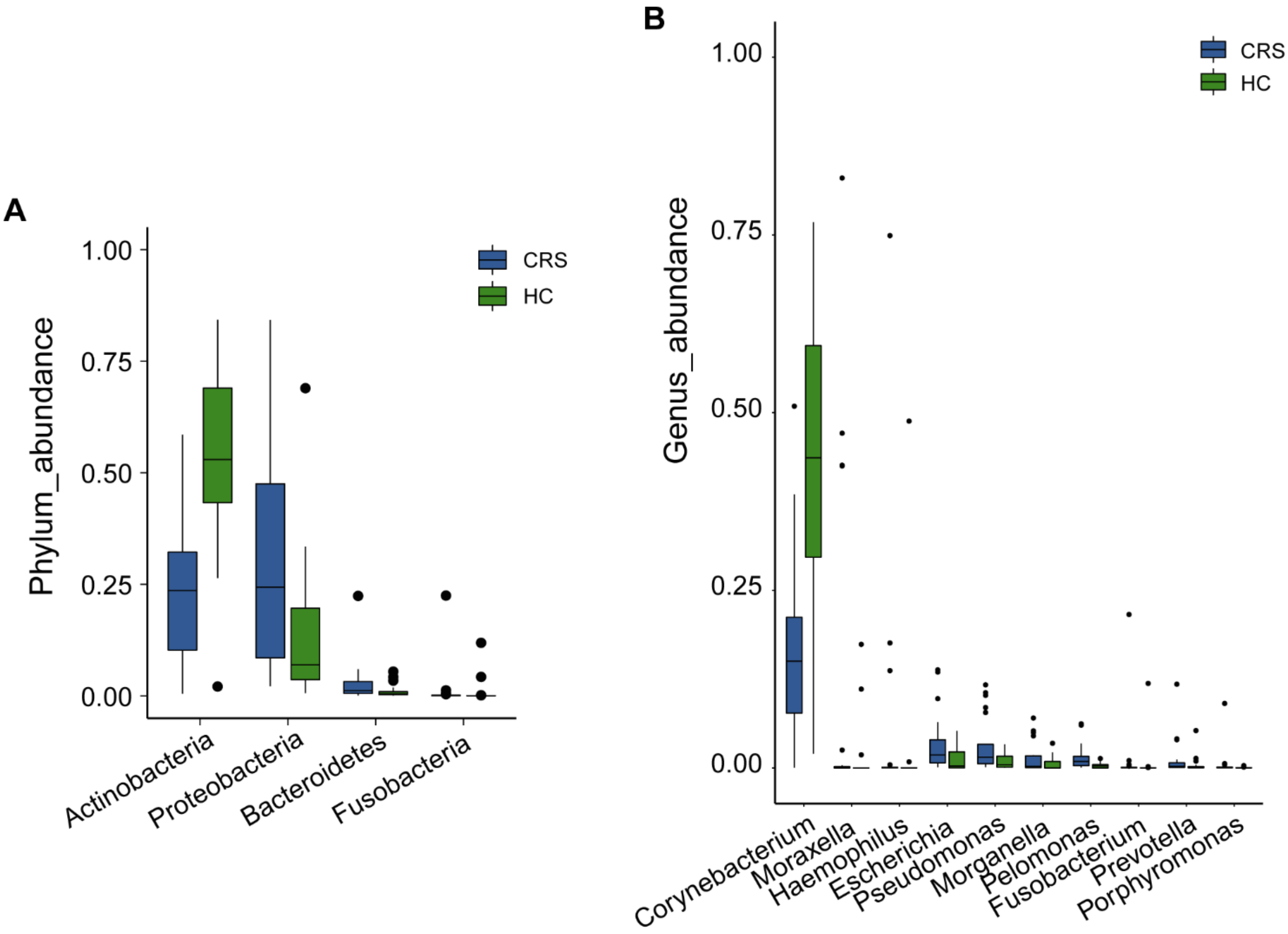

Figure S4 Comparison of the nasal microbial composition at the (A) phylum and (B) genus levels between the CRS and HC groups (all $P < 0.05$). Abbreviations: CRS, chronic rhinosinusitis; HC, healthy control.
