# Supplementary material for: Characteristics and Clinical Implications of the Nasal Microbiota in Extranodal NK/T-Cell Lymphoma, Nasal Type
Source: Front Cell Infect Microbiol. 2021 Sep 10;11:686595. doi: 10.3389/fcimb.2021.686595 (PMC8461088; doi:10.3389/fcimb.2021.686595)
Supplement: Supplementary file 6 [file Image_5.pdf]

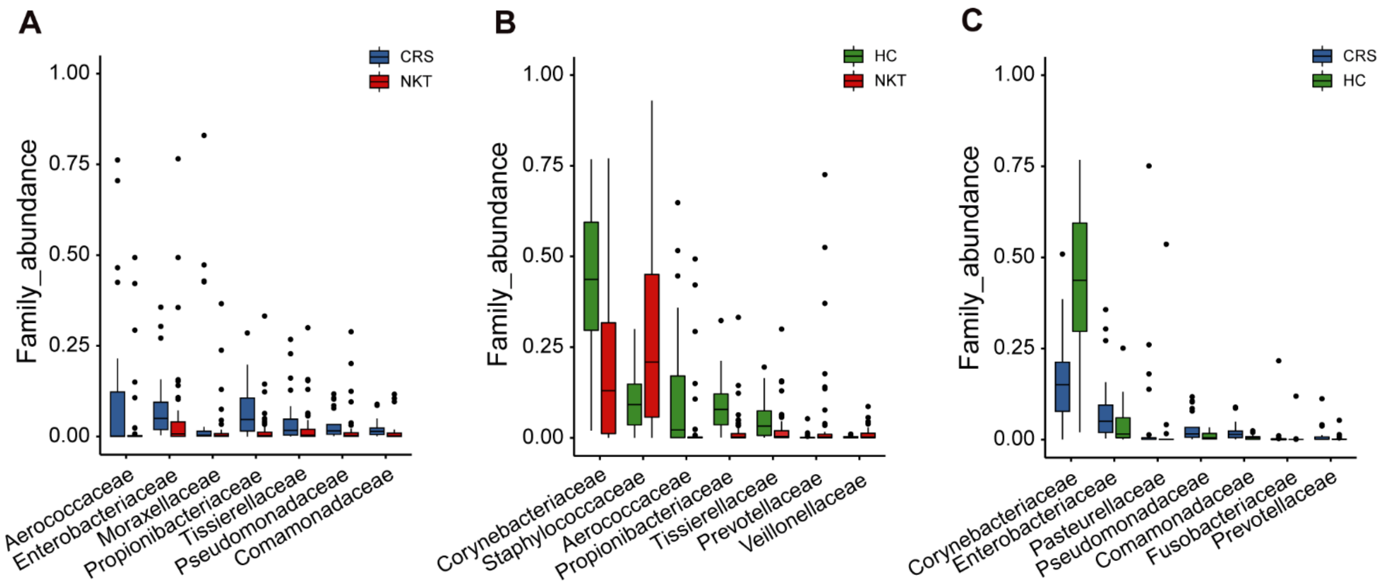

Figure S5 Comparison of the nasal microbial composition at the family level between the (A) CRS and NKT groups, (B) HC and NKT groups, and (C) CRS and HC groups (all $P < 0.05$). Abbreviations: NKT, natural killer/T cell lymphoma; CRS, chronic rhinosinusitis; HC, healthy control.
