# Supplementary material for: Characteristics and Clinical Implications of the Nasal Microbiota in Extranodal NK/T-Cell Lymphoma, Nasal Type
Source: Front Cell Infect Microbiol. 2021 Sep 10;11:686595. doi: 10.3389/fcimb.2021.686595 (PMC8461088; doi:10.3389/fcimb.2021.686595)
Supplement: Supplementary file 7 [file Image_6.pdf]

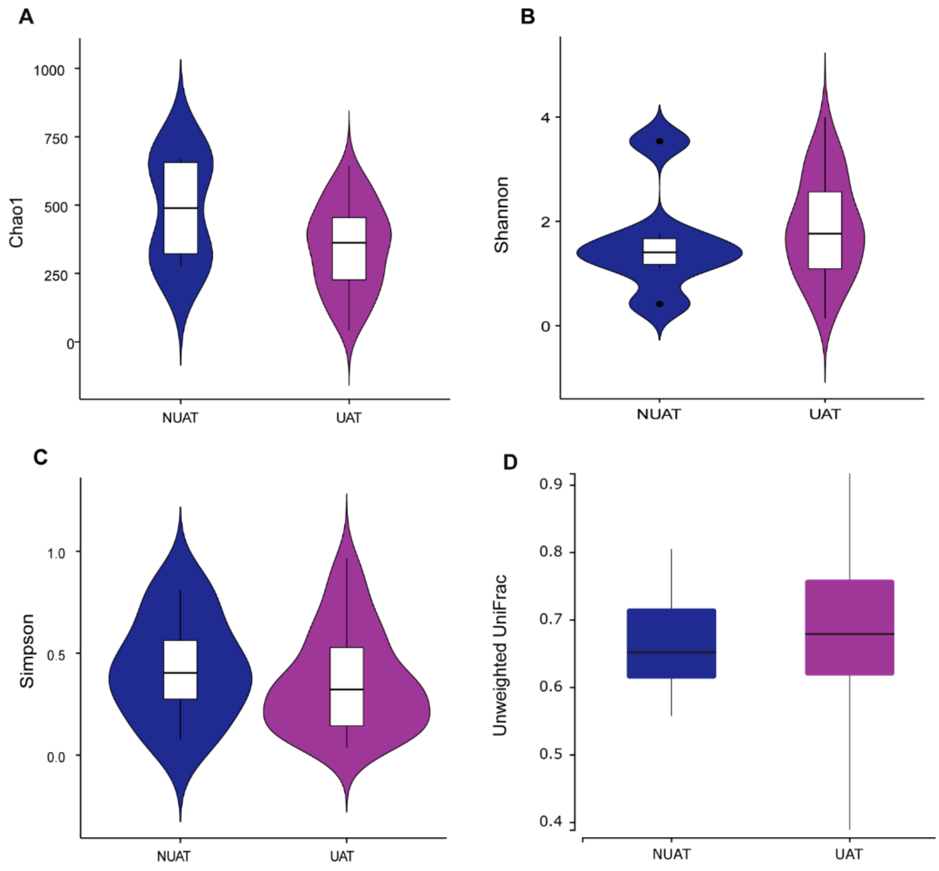

Figure S6 Comparison of the microbial alpha diversity by (A) Chao1 index, *P* = 0.10777, (B) Shannon index, *P* = 0.49281, (C) Simpson index, *P* = 0.5133, Wilcox Test; and (D) beta diversity, *P* = 0.34641, unweighted UniFrac index, between the NUAT and UAT groups. Abbreviations: NUAT, non-upper aerodigestive tract; UAT, upper aerodigestive tract.
