# Supplementary material for: Characteristics and Clinical Implications of the Nasal Microbiota in Extranodal NK/T-Cell Lymphoma, Nasal Type
Source: Front Cell Infect Microbiol. 2021 Sep 10;11:686595. doi: 10.3389/fcimb.2021.686595 (PMC8461088; doi:10.3389/fcimb.2021.686595)
Supplement: Supplementary file 8 [file Image_7.pdf]

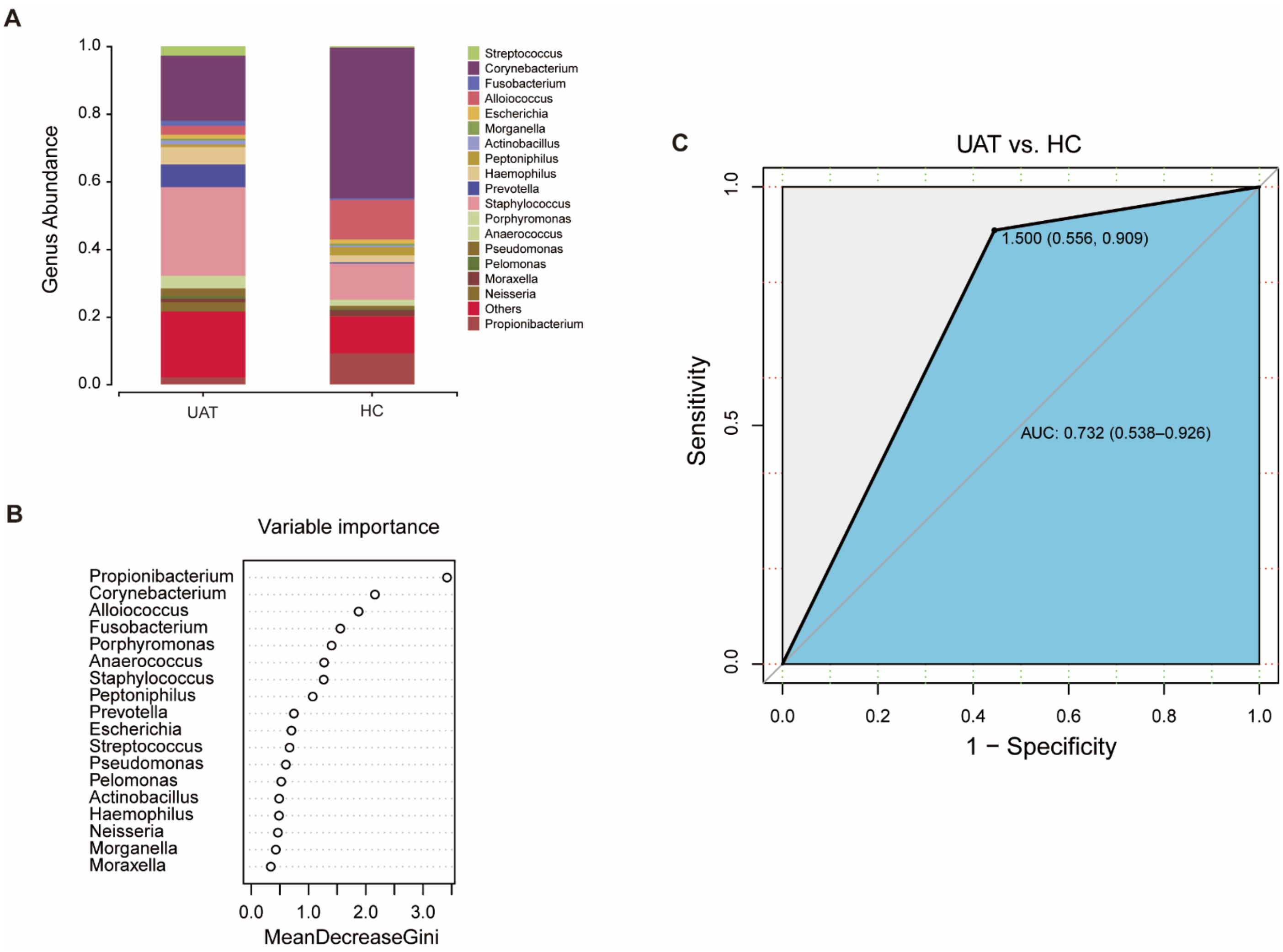

Figure S7 (A) Composition of the nasal microbiota at the genus level between the UAT and HC groups (only genera with relative abundances > 0.5% are shown), and (B) these taxa are ranked from the top to bottom by decreasing Gini index scores determined from the random forest algorithm trained to distinguish the 2 cohorts. Importantly, these genus markers in combination might be used to distinguish UAT-NKTCL from HC, (C) achieving an AUC of 0.732 (95% CI: 0.538 to 0.926). Abbreviations: UAT, upper aerodigestive tract; HC, healthy control; AUC, area under the curve; 95% CI, 95% confidence interval.
