# Supplementary material for: Characteristics and Clinical Implications of the Nasal Microbiota in Extranodal NK/T-Cell Lymphoma, Nasal Type
Source: Front Cell Infect Microbiol. 2021 Sep 10;11:686595. doi: 10.3389/fcimb.2021.686595 (PMC8461088; doi:10.3389/fcimb.2021.686595)
Supplement: Supplementary file 10 [file Image_9.pdf]

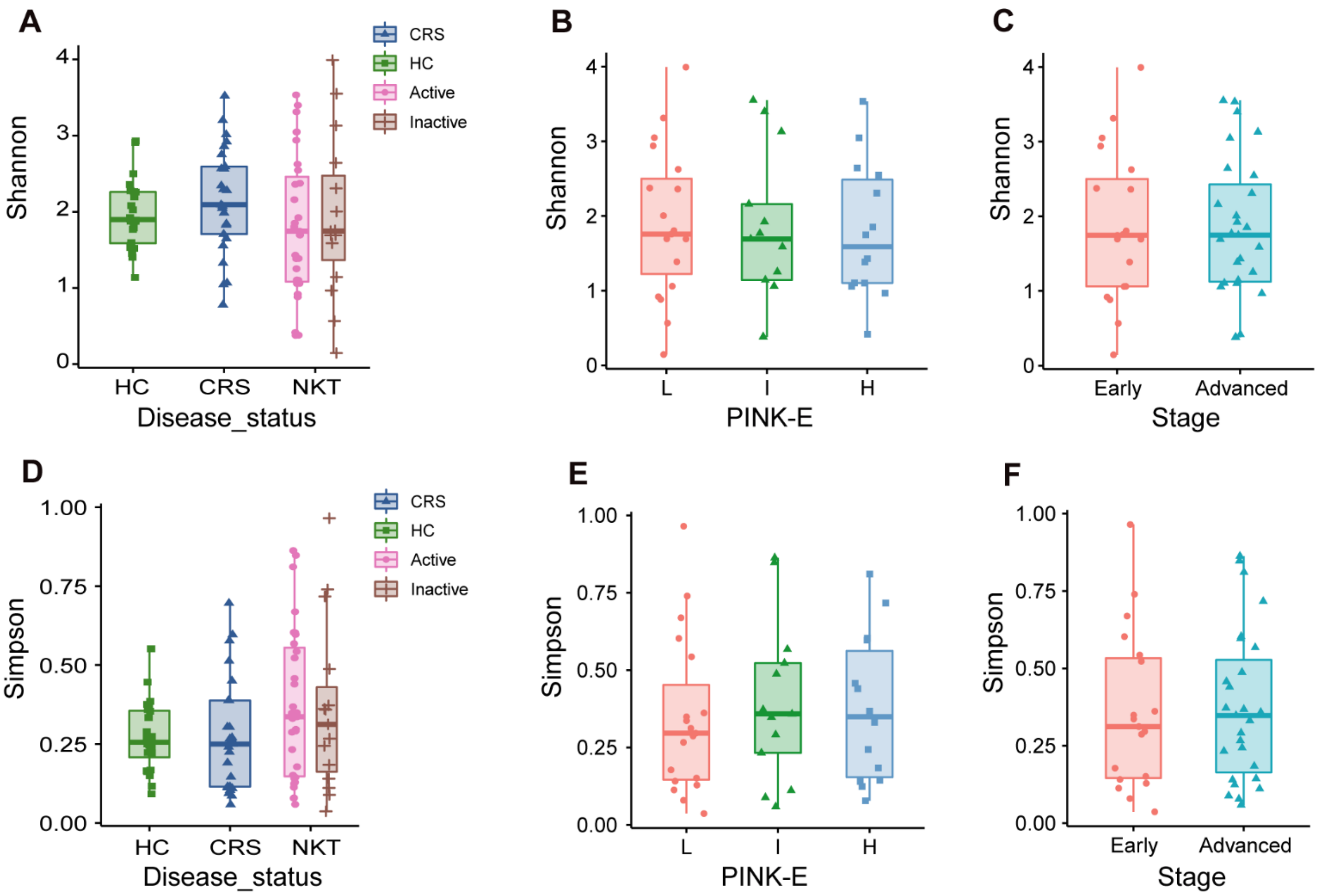

Figure S9 Associations of the nasal microbiota with disease status and prognosis indicators of NKTCL. Alpha diversity of the nasal microbiota, as measured by the (A-C) Shannon index, and (D-F) Simpson index, in active and inactive status; low risk, intermediate risk, and high risk based on PINK-E; and early and advanced stage NKTCL patients, with no significant difference between groups. Abbreviations: HC, healthy control; CRS, chronic rhinosinusitis; NKT, natural killer/T cell lymphoma; L, low risk; I, intermediate risk; H, high risk; PINK-E, prognostic index for natural killer lymphoma-Epstein-Barr virus.
