# Supplementary material for: Characteristics and Clinical Implications of the Nasal Microbiota in Extranodal NK/T-Cell Lymphoma, Nasal Type
Source: Front Cell Infect Microbiol. 2021 Sep 10;11:686595. doi: 10.3389/fcimb.2021.686595 (PMC8461088; doi:10.3389/fcimb.2021.686595)
Supplement: Supplementary file 11 [file Table_1.pdf]

Table S1 Participant characteristics at the time of nasal sampling.

	NKT (n=46)	CRS (n=25)	HC (n=24)	P values (CRS vs. NKT)	P values (HC vs. NKT)
Age	43.0	36.0	40.5	0.091	0.096
median years (min-max)	(18-74)	(18-64)	(20-65)		
Gender					
Female	16 (34.8%)	8 (32.0%)	8 (33.3%)	1.000	1.000
Male	30 (65.2%)	17 (68.0%)	16 (66.7%)		
Ever smoker					
No	30 (65.2%)	19 (76.0%)	16 (66.7%)	0.427	1.000
Yes	16 (34.8%)	6 (24.0%)	8 (33.3%)		
BMI					
median kg/ m^2 (min-max)	22.76 (15.56-31.96)	24.91 (17.58-31.83)	23.00 (16.76-27.74)	0.053	0.833
Comorbidities					
Asthma	1 (2.2%)	1 (4.0%)	0		
Diabetes	4 (8.7%)	0	0		
Hepatitis B	5 (11.1%)	0	0		
Hypertension	4 (8.7%)	0	0		
Primary_site					
NUAT	6 (13.0%)				
UAT	40 (87.0%)				
B_symptoms					
Absence	26 (56.5%)				
Presence	20 (43.5%)				
Stage					
I/II	19 (41.3%)				
III/IV	27 (58.7%)				
EBV_level					
Increased	16 (34.8%)				
Normal	30 (65.2%)				
LN involvement					
No	23 (50.0%)				
Yes	23 (50.0%)				
PINK-E					
Low risk	19 (41.3%)				
Intermediate risk	13 (28.3%)				
High risk	14 (30.4%)				
Serum_LDH					
Increased	19 (41.3%)				
Normal	27 (58.7%)				
ECOG performance status					
0~1	38 (82.6%)				
≥2	8 (17.4%)				
BM involvement					
No	42 (91.3%)				
Yes	4 (8.7%)				
History treatment					
Newly diagnosed	19 (41.3%)				
Chemotherapy_history	27 (58.7%)				
Radiotherapy_history	10 (21.7%)				

Abbreviations: BMI, body mass index; NUAT, Non-upper aerodigestive tract; UAT, Upper aerodigestive tract; EBV, *Epstein-Barr* virus; LN, lymph node; PINK-E, Prognostic index for natural killer lymphoma-Epstein-Barr virus; LDH, Lactate dehydrogenase; ECOG, Eastern Cooperative Oncology Group; BM, bone marrow; NKT, natural killer T-cell lymphoma; CRS, Chronic rhinosinusitis; HC, Health control. Continuous variables were compared using Kruskal-Wallis rank sum test between both groups. Fisher's exact test compared categorical variables.
