# Supplementary material for: Characteristics and Clinical Implications of the Nasal Microbiota in Extranodal NK/T-Cell Lymphoma, Nasal Type
Source: Front Cell Infect Microbiol. 2021 Sep 10;11:686595. doi: 10.3389/fcimb.2021.686595 (PMC8461088; doi:10.3389/fcimb.2021.686595)
Supplement: Supplementary file 12 [file Table_2.pdf]

Table S2 Significantly different genera between the NKT and CRS groups.

Genus	NKT (%)	CRS (%)	P value
Alloiococcus	3.309354	12.096886	0.003161
Moraxella	1.080967	8.739392	0.001022
Propionibacterium	2.238812	7.097638	1.26E-4
Pseudomonas	2.053138	3.009524	3.11E-4
Escherichia	1.039929	3.339508	1.6E-5
Anaerococcus	1.233413	2.088798	0.044322
Peptoniphilus	0.661954	1.974668	0.001569
Pelomonas	0.734055	1.411979	5.9E-5
Morganella	0.39784	1.294429	1.39E-4
Finegoldia	0.334868	0.833967	0.020634

Abbreviations: NKT, natural killer/T cell lymphoma; CRS, chronic rhinosinusitis.
