# Supplementary material for: Characteristics and Clinical Implications of the Nasal Microbiota in Extranodal NK/T-Cell Lymphoma, Nasal Type
Source: Front Cell Infect Microbiol. 2021 Sep 10;11:686595. doi: 10.3389/fcimb.2021.686595 (PMC8461088; doi:10.3389/fcimb.2021.686595)
Supplement: Supplementary file 13 [file Table_3.pdf]

Table S3 Significantly different genera between the NKT and HC groups.

Genus	HC (%)	NKT (%)	P value
Corynebacterium	44.534592	19.126466	2.1E-5
Alloiococcus	11.527495	3.309354	1.51E-4
Propionibacterium	9.16737	2.238812	1.0E-6
Prevotella	0.405364	5.773325	0.028076
Peptoniphilus	2.507551	0.661954	1.71E-4
Anaerococcus	1.817373	1.233413	0.020642
Staphylococcus	10.725505	29.561843	0.016104

Abbreviations: NKT, natural killer/T cell lymphoma; HC, healthy control.
