# Supplementary material for: Characteristics and Clinical Implications of the Nasal Microbiota in Extranodal NK/T-Cell Lymphoma, Nasal Type
Source: Front Cell Infect Microbiol. 2021 Sep 10;11:686595. doi: 10.3389/fcimb.2021.686595 (PMC8461088; doi:10.3389/fcimb.2021.686595)
Supplement: Supplementary file 14 [file Table_4.pdf]

Table S4.1 Significantly different phyla between the CRS and HC groups.

Phylum	HC (%)	CRS (%)	P value
Actinobacteria	54.424764	23.675013	4.0E-6
Proteobacteria	14.031263	30.537551	0.007585
Bacteroidetes	1.026975	2.662182	0.025747
Fusobacteria	0.709439	1.065055	0.044431

Abbreviations: CRS, chronic rhinosinusitis; HC, healthy control.

Table S4.2 Significantly different genera between the CRS and HC groups.

Genus	HC (%)	CRS (%)	P value
Corynebacterium	44.534592	15.939001	2.0E-6
Moraxella	1.728566	8.739392	0.02956
Haemophilus	2.083416	4.31686	0.008017
Escherichia	1.234903	3.339508	0.002612
Pseudomonas	0.936959	3.009524	0.006728
Morganella	0.579416	1.294429	0.026462
Pelomonas	0.348213	1.411979	5.61E-4
Fusobacterium	0.524732	0.975071	0.019806
Prevotella	0.405364	1.082856	0.033172
Porphyromonas	0.051914	0.462737	0.030768

Abbreviations: CRS, chronic rhinosinusitis; HC, healthy control.
