# Supplementary material for: Characteristics and Clinical Implications of the Nasal Microbiota in Extranodal NK/T-Cell Lymphoma, Nasal Type
Source: Front Cell Infect Microbiol. 2021 Sep 10;11:686595. doi: 10.3389/fcimb.2021.686595 (PMC8461088; doi:10.3389/fcimb.2021.686595)
Supplement: Supplementary file 15 [file Table_5.pdf]

Table S5.1 Significantly different families between the NKT and CRS groups.

Family	NKT (%)	CRS (%)	P value
Aerococcaceae	3.374063	12.228736	0.031629
Enterobacteriaceae	5.890942	8.599533	7.33E-04
Moraxellaceae	2.209084	9.084347	0.036735
Propionibacteriaceae	2.256561	7.111442	1.09E-04
Tissierellaceae	2.73455	4.938265	0.049033
Pseudomonadaceae	2.058859	3.01783	3.42E-04
Comamonadaceae	1.110698	2.055997	1.53E-04

Abbreviations: NKT, natural killer/T cell lymphoma; CRS, chronic rhinosinusitis.

Table S5.2 Significantly different families between the NKT and HC groups.

Family	NKT (%)	HC (%)	P value
Corynebacteriaceae	19.126466	44.534592	2.10E-05
Staphylococcaceae	29.59948	10.7581	0.016104
Aerococcaceae	3.374063	11.552503	0.002586
Propionibacteriaceae	2.256561	9.173407	1.00E-06
Tissierellaceae	2.73455	5.199942	0.002923
Prevotellaceae	5.141077	0.386721	0.027203
Veillonellaceae	1.063848	0.229474	0.048438

Abbreviations: NKT, natural killer/T cell lymphoma; HC, healthy control.

Table S5.3 Significantly different families between the CRS and HC groups.

Family	CRS (%)	HC (%)	P value
Corynebacteriaceae	15.939001	44.534592	2.00E-06
Enterobacteriaceae	8.599533	4.206311	0.027105
Pasteurellaceae	5.493538	2.516174	0.008289
Pseudomonadaceae	3.01783	0.939623	0.006333
Comamonadaceae	2.055997	0.628988	0.001748
Fusobacteriaceae	0.975071	0.524732	0.019806
Prevotellaceae	1.008419	0.386721	0.033172

Abbreviations: CRS, chronic rhinosinusitis; HC, healthy control.
