# Supplementary material for: Characteristics and Clinical Implications of the Nasal Microbiota in Extranodal NK/T-Cell Lymphoma, Nasal Type
Source: Front Cell Infect Microbiol. 2021 Sep 10;11:686595. doi: 10.3389/fcimb.2021.686595 (PMC8461088; doi:10.3389/fcimb.2021.686595)
Supplement: Supplementary file 16 [file Table_6.pdf]

Table S6 Significantly different function prediction of microbial gene between the NKT and HC groups.

Function	NKT (%)	HC (%)	P value	FDR
Proteasome	0.01191	0.035871	1.0E-6	1.84E-4
Epithelial cell signaling in *Helicobacter pylori* infection	0.149458	0.100613	5.0E-6	3.07E-4
Styrene degradation	0.145402	0.279251	3.1E-5	0.001426
Meiosis-yeast	0.004818	0.009653	4.8E-5	0.001735
RNA transport	0.048616	0.037003	5.9E-5	0.001735
Thiamine metabolism	1.387198	1.582751	6.6E-5	0.001735
Base excision repair	0.948013	1.050438	1.73E-4	0.003979
Ribosome biogenesis in eukaryotes	0.064155	0.076779	3.25E-4	0.006273
African trypanosomiasis	0.019566	0.037198	3.75E-4	0.006273
Caprolactam degradation	0.143739	0.223797	3.75E-4	0.006273
Dioxin degradation	0.33619	0.226124	6.83E-4	0.010473
Selenocompound metabolism	1.048683	1.123821	0.001065	0.013724
Sulfur relay system	1.36098	1.496389	0.001113	0.013724
Bisphenol degradation	0.099613	0.167266	0.001158	0.013724
Phenylalanine metabolism	0.30302	0.36194	0.001214	0.013724
Oxidative phosphorylation	0.42325	0.487567	0.001268	0.013724
Nicotinate and nicotinamide metabolism	1.015149	1.091558	0.003297	0.033703
Aminobenzoate degradation	0.255347	0.291803	0.004018	0.038911
Drug metabolism-other enzymes	1.089251	1.239339	0.004516	0.041547

Abbreviations: NKT, natural killer/T cell lymphoma; HC, healthy control.
