# Supplementary material for: Characteristics and Clinical Implications of the Nasal Microbiota in Extranodal NK/T-Cell Lymphoma, Nasal Type
Source: Front Cell Infect Microbiol. 2021 Sep 10;11:686595. doi: 10.3389/fcimb.2021.686595 (PMC8461088; doi:10.3389/fcimb.2021.686595)
Supplement: Supplementary file 17 [file Table_7.pdf]

Table S7 Significantly different function prediction of microbial genes between the NKT and CRS groups.

Function	NKT (%)	CRS (%)	P value	FDR
Glutathione metabolism	0.763921	0.869273	0.001392	0.063686
Styrene degradation	0.145402	0.255739	0.001392	0.063686
Geraniol degradation	0.46807	0.618677	8.0E-4	0.063686
Biosynthesis of unsaturated fatty acids	0.356499	0.441929	0.001712	0.063686
Caprolactam degradation	0.143739	0.233053	0.004089	0.095025
Plant-pathogen interaction	0.151157	0.172708	0.004412	0.095025
Epithelial cell signaling in *Helicobacter pylori* infection	0.149458	0.121757	0.003788	0.095025
Pantothenate and CoA biosynthesis	1.656851	1.596917	0.005522	0.102709
Phenylalanine metabolism	0.30302	0.360832	0.006396	0.102827
Lysine biosynthesis	1.399947	1.332866	0.006634	0.102827
Bacterial secretion system	0.823664	0.896595	0.007942	0.113632
Bacterial chemotaxis	0.39699	0.5049	0.008835	0.117379
Fluorobenzoate degradation	0.069036	0.085504	0.012486	0.14136
Methane metabolism	0.410888	0.43812	0.01292	0.14136
Non-homologous end-joining	0.012149	0.017916	0.012489	0.14136
Flagellar assembly	0.183764	0.262085	0.019206	0.188017
Oxidative phosphorylation	0.42325	0.474963	0.021828	0.190481
Glyoxylate and dicarboxylate metabolism	0.595622	0.662577	0.02253	0.190481
Toluene degradation	0.416407	0.498494	0.027156	0.219609
Proteasome	0.01191	0.017344	0.031643	0.245233
Steroid hormone biosynthesis	0.009882	0.007955	0.033601	0.249991
Nitrotoluene degradation	0.067352	0.095828	0.037833	0.270651
Polycyclic aromatic hydrocarbon degradation	0.013233	0.013684	0.044874	0.309132
Carotenoid biosynthesis	0.241494	0.156688	0.049033	0.325719

Abbreviations: NKT, natural killer/T cell lymphoma; CRS, chronic rhinosinusitis.
